# Beyond Lithium-Based Batteries

**DOI:** 10.3390/ma13020425

**Published:** 2020-01-16

**Authors:** Jasper Biemolt, Peter Jungbacker, Tess van Teijlingen, Ning Yan, Gadi Rothenberg

**Affiliations:** 1 Van ’t Hoff Institute for Molecular Sciences, University of Amsterdam, Science Park 904, 1098XH Amsterdam, The Netherlands; 2 School of Physics and Technology, Wuhan University, No.299 Bayi Rd. Wuhan 430072, China

**Keywords:** electrochemistry, redox reaction, energy storage, rechargeable batteries, sodium, sulfur, PEST analysis, supercapacitors, circular economy, low-carbon technologies

## Abstract

We discuss the latest developments in alternative battery systems based on sodium, magnesium, zinc and aluminum. In each case, we categorize the individual metals by the overarching cathode material type, focusing on the energy storage mechanism. Specifically, sodium-ion batteries are the closest in technology and chemistry to today’s lithium-ion batteries. This lowers the technology transition barrier in the short term, but their low specific capacity creates a long-term problem. The lower reactivity of magnesium makes pure Mg metal anodes much safer than alkali ones. However, these are still reactive enough to be deactivated over time. Alloying magnesium with different metals can solve this problem. Combining this with different cathodes gives good specific capacities, but with a lower voltage (<1.3 V, compared with 3.8 V for Li-ion batteries). Zinc has the lowest theoretical specific capacity, but zinc metal anodes are so stable that they can be used without alterations. This results in comparable capacities to the other materials and can be immediately used in systems where weight is not a problem. Theoretically, aluminum is the most promising alternative, with its high specific capacity thanks to its three-electron redox reaction. However, the trade-off between stability and specific capacity is a problem. After analyzing each option separately, we compare them all via a political, economic, socio-cultural and technological (PEST) analysis. The review concludes with recommendations for future applications in the mobile and stationary power sectors.

## 1. Introduction

Our society depends on electricity, which powers our homes, jobs, hobbies, transportation and communication. We even have electricity on the go, which is essential as more and more people use mobile devices. Batteries have become a crucial component of our daily lives. Yet, unlike the power outlet at our homes that seemingly supplies an infinite amount of energy (as long as we pay the bills), batteries can only hold a limited amount of energy. So, how do these batteries store and release electrical energy? Indeed, the importance of this question is reflected in the awarding of the 2019 Nobel Prize in Chemistry to Stanley Whittingham, John Goodenough and Akira Yoshino for their research and development of lithium-ion batteries [1].

**Figure 1:**
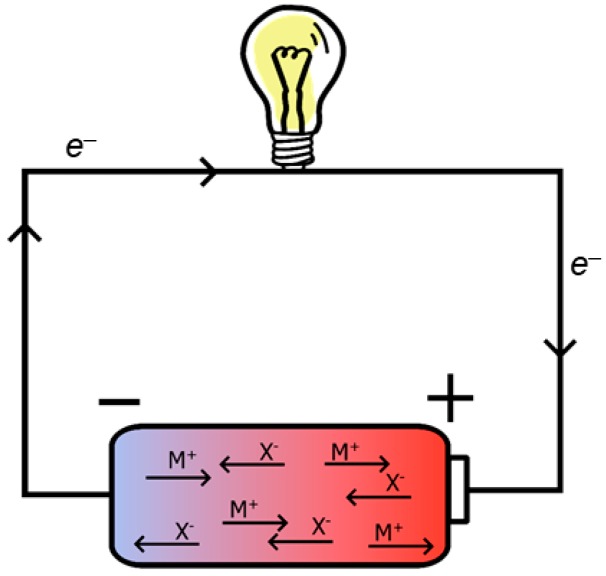
Drawing of a traditional battery and the flow of electrons (e−), cations (M+) and anions (X).

Figure 1 shows a circuit with a battery powering a lamp. Every battery has an anode and a cathode. The anode supplies electrons via an oxidation reaction. These move through the circuit to the cathode. Here, the electrons are stored again via a reduction reaction. The energy supplied by the electric current comes from the difference in energy of the redox reactions, also referred to as the potential, and the number of electrons that are flowing, referred to as the charge. A battery is empty when the redox reaction ends and the flow of electrons stops. In a rechargeable battery, an external voltage can reverse the electron flow via the reverse reactions.

**Figure 2:**
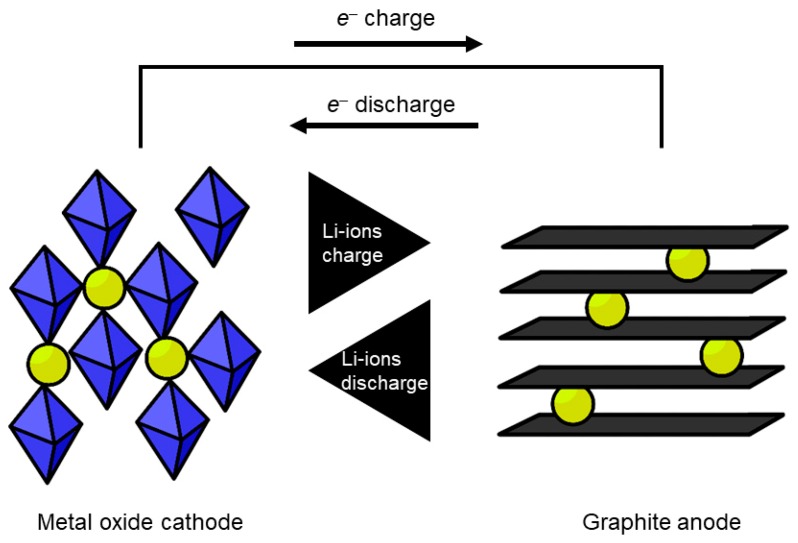
The ion flow in lithium-ion battery: When the cell charges and discharges, ions shuttle between cathode (positive electrode) and anode (negative electrode). On discharge, the anode undergoes oxidation, or loss of electrons, and the cathode sees a reduction, or a gain of electrons. During charge, the movements are reversed.

In a lithium-ion battery, the anode consists of a carbon material (commonly graphite) with lithium stored between the carbon layers [2]. These layers stabilize the lithium in a high energy metallic state. When discharging, the lithium metal dissociates into a lithium cation and an electron, see Equation (1). The Li cation travels through the electrolyte (a liquid medium with a separator) to the cathode, while the electron travels through the external circuit (Figure 2). The separator allows ions to move through but blocks the electrons. This forces the electrons to move via a different route and avoids self-discharge (the discharging of a battery without powering a device). At the cathode, the electron reduces the cathode material (often CoO_2_, as in Equation (2)) and an alloy with the lithium is formed [3].

Good battery anodes and cathodes need certain characteristics. To explain these, we use the Li/C and CoO_2_ type anode and cathode as examples. The main characteristic of the anode is its ability to reduce the Li^+^ (or other metals for different batteries) cations during charging. In the Li/C anode, this occurs by forming metallic Li between the graphite sheets, generating high-energy yet stabilized states of the metallic Li. At the same time, during discharge, all the Li^0^ atoms must be able to oxidize to Li^+^ cations without significant energy loss between the reduction (charging) and oxidation (discharging). For cathodes, an important aspect is the incorporation of electrons liberated during discharge into the anode material (and vice versa during charging). This occurs in CoO_2_ by the reduction of Co^4^ to Co^3+^, while Li^+^ cations counterbalance the additional negative charge in the cathode. However, it is not necessary that the cations generated at the anode counteract the negative charge in the cathode. Besides these individual characteristics, both the anode and cathode must remain stable during the charging and discharging, and the corresponding redox reactions must be completely reversible [4].

(1) $$\mathrm{Li}\to {\mathrm{Li}}^{+}+e^{-}$$

(2) $${\mathrm{Li}}^{+}+e^{-}+{\mathrm{CoO}}_{2}\to {\mathrm{LiCoO}}_{2}$$

The redox reactions in a battery determine its voltage. For the lithium-ion battery, the two redox couples are Li/Li^+^ and Co^4+^/Co^3+^. This gives a theoretical voltage of 6.94 V when using the pure metals [5]. However, these two theoretical redox pairs do not represent the real Li/C anode and CoO_2_ cathode, which together result in a voltage of 3.8 V. This voltage is an important descriptor of batteries, as it shows the amount of energy stored in the electrons. Other frequently used descriptors are the energy density and power density. Energy density is the amount of energy in a given mass (or volume) and power density is the amount of power in a given mass [6]. The difference between the two can be described with a bottle as an example. For the bottle, the energy density is the volume of liquid it can hold, while the power density tells how fast the bottle will empty/fill. Thus, in a battery, a high energy density translates to a high amount of stored energy in a small amount of mass. A high power density shows that a large amount of energy can be released in a small timeframe. For battery research, the term energy density can be misleading, since it includes the voltage. Since batteries can operate at different voltages, specific capacity is used, an expression for the amount of electrical charge stored per mass unit.

The problem with lithium batteries is that today’s batteries are close to the maximum theoretical capacity, when using the Li-C anode (370 mA g^−1^) [7,8,9,10,11,12]. As an alternative, lithium-sulfur (Li-S) batteries have a high theoretical capacity because of the sulfur cathode [13]. However, these are less stable and have a low cycle stability compared to the metal-oxide cathodes. Furthermore, using the high capacity of the sulfur requires anodes with higher energy densities, such as pure lithium (3861 mAh g^−1^) [14] but these come at the cost of safety. Current Li-C batteries already showed thermal runaways, with disastrous results [15,16,17]. For a detailed account of the causes of thermal runaway incidents with Li-ion batteries see Feng et al. [18] These safety issues will only increase further when using pure lithium anodes. Accordingly, many groups study Li-ion batteries to counteract the capacity limit and safety issues. Recent discoveries in Li-based batteries are reviewed elsewhere [19,20,21,22,23].

**Figure 3:**
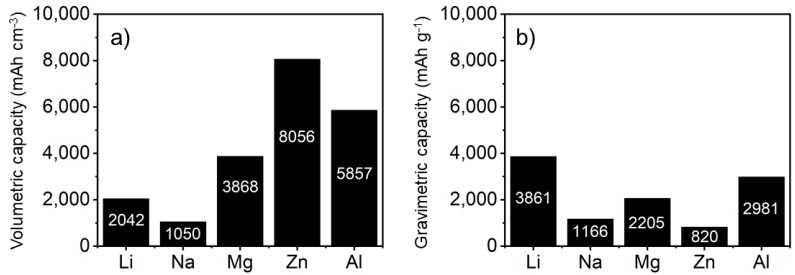
Comparison of volumetric capacity (**a**) and gravimetric capacity (**b**) of different metals [14].

In addition to safety concerns, the depletion of the lithium reserves and the corresponding price increase the urge for alternative battery types [24]. Thus, sodium (Na), zinc (Zn), magnesium (Mg) and aluminum (Al) battery systems are being explored as alternative candidates due to their low cost and high abundance [14]. Figure 3 compares the volumetric/gravimetric capacities for lithium and some potential alternatives. From these diagrams we see that Mg, Al and Zn are promising alternatives for lithium. Potassium-based batteries are also a viable option, but this research area is still in its infancy [25]. Furthermore, potassium suffers from slow diffusion kinetics in solids (diffusion within anodes and cathodes) and its high atomic mass yields even lower gravimetric capacities then sodium. Due to the combination of the stage of the research and the inherent drawbacks, potassium-based batteries are excluded from this review.

In this review, we discuss some alternative systems based on Na, Zn, Mg and Al, following the literature published in 2017–2019. Despite this short period, the number of publications on this topic is large. Metal-air batteries are excluded, since these fall outside of the scope. We categorize the individual metals by the overarching cathode material type and focus on the energy storage mechanism and the latest developments. Then, we compare the different alternatives using a PEST analysis, which covers political, economic, socio-cultural and technological aspects. The review concludes with a cross-system comparison and recommendations.

## 2. Sodium-Ion Batteries

From a fundamental point, sodium is the most straightforward alternative for lithium. The chemistry is highly similar and current technologies are compatible with sodium ions [26,27], while sodium is more abundant and cheaper than lithium [26]. The redox potential of sodium is also highly similar to that of lithium ($E_{{\mathrm{Na}}^{+}/\mathrm{Na}}$ = −2.71 V vs. $E_{{\mathrm{Li}}^{+}/\mathrm{Li}}$ = −3.04 V) resulting in similar battery voltages. The main drawback is the lower gravimetric capacity of sodium compared to lithium (see Figure 3). Sodium and lithium both release one electron when forming the ion, but sodium is three times heavier. This makes sodium an unlikely candidate to replace lithium in devices where the overall weight is important. However, the lower cost and higher abundance makes sodium appealing for large grid storage applications.

While most current Li-ion techniques are compatible with sodium, the graphite-based anode is not. This is attributed to both the large ionic radius of sodium (1.02 Å) and the weak chemical bonds between Na-ions and carbon materials [28,29]. This field, therefore, researches both anode and cathode materials. However, the current stage of anode development uses new anode materials as a cathode against pure sodium. These tests are highly interesting to determine the sodium uptake of the material but do not provide an accurate reference point for battery applications. We therefore disregard these papers in the current review.

The research on Na-ion batteries is booming, with ~1400 papers published in the last year alone. Below we extract the main topics and points. Readers interested in more detail are referred to specific overviews on Na-ion batteries [29,30,31,32,33,34,35,36,37,38,39,40,41,42,43].

### 2.1. Metal Oxides as Cathodes in Na-Ion Batteries

The most researched materials for Na-ion battery cathodes are layered structured oxides (NaMO_2_, where M = Co, Ni, Fe, Ti, Cr or a mixture of two or more transition metals) [44,45,46,47,48,49]. This is mainly attributed to current Li-ion batteries also using these types of cathodes. Metal oxides generally exhibit weak interlayer interactions and a vacant 2D space for guest ion diffusion [41]. These properties can be attributed to the layered crystal structure of the metal oxides. Such oxides are made up of edge-sharing transition metal centered oxygen octahedral (MO_6_).

Na^+^ ions can intercalate between the layers into the trigonal prismatic or the octahedral vacancies. During the discharge, the transition metals are reduced, while the Na^+^ intercalates into the material, as in Equation (4) [50]. This results in multiple phase transformations due to the ordering of Na^+^ ions within the metal oxide lattice. The reaction at the cathode is the same as with lithium [3]. During discharge, the Na^+^ ions diffuse into the cathode lattice. Co^4+^ is reduced to Co^3+^ to compensate for the change in charge. This means that the overall reaction involves the Na/Na^+^ and the Co^4+^/Co^3+^ redox couples, of which the composites deliver an actual 3.5 V [51]. This mechanism is also observed for the other metal oxides and the cathodes show good cycling stability. However, the larger size of sodium increases the volume expansion during intercalation, resulting in stability risks in real-life applications [52]. Alloys of different metal oxides or the formation of composite materials can diminish the effects. Excellent results were obtained, for example, with Na_x_MnO_2_ type materials, which show capacities up to 200 mAh g^−1^ [53]. The Na^+^ ions can be reversibly de/intercalated into the material with good capacity retention.

(3) $$\mathrm{Na}\to {\mathrm{Na}}^{+}+e^{-}$$

(4) $${\mathrm{Na}}^{+}+e^{-}+{\mathrm{CoO}}_{2}\to {\mathrm{NaCoO}}_{2}$$

Overall, layered metal oxides are promising cathode materials for Na-ion batteries, especially as these materials are already used for Li-ion batteries. They can deliver high voltages and capacities are decent, making them a potential candidate for large grid storage. Furthermore, some research has explored the possibility of using these materials as anodes [41,53,54,55]. The disadvantage of this method is the already lowered redox potential of the Na in these materials, which will result in low operating voltages.

### 2.2. Polyanion Compounds as Cathodes in Na-Ion Batteries

Polyanion compounds comprise a transition metal that is ionically bound to tetrahedron anion units ((XO_4_)^n−^, where X = S, P, Si, W, As, or Mo) [41]. Na_3_V_2_(PO_4_)_3_ has been studied extensively in this class of compounds and serves as a reference point [35,56,57]. During charging/discharging, the structure changes between Na_3_V_2_(PO_4_)_3_ and NaV_2_(PO_4_)_3_ via a two-phase reaction. This reversible (de)sodiation occurs similarly to the metal oxides via a transition metal redox couple. In this case, the conversions between V^3+^ ↔ V^4+^ and is represented by Equation (5).

(5) $${\mathrm{NaV}}_{2}{\left({\mathrm{PO}}_{4}\right)}_{3}+2{\mathrm{Na}}^{+}+2e^{-}\to {\mathrm{Na}}_{3}V_{2}{\left({\mathrm{PO}}_{4}\right)}_{3}$$

As mentioned, the reaction occurs in a two-step process, with Na_2_V_2_(PO_4_)_3_ as the intermediate, which contains both V^3+^ and V^4+^. The overall reaction in a battery involves the Na/Na^+^ and V^4+^/V^3+^ redox couples, giving a cell has a voltage 3.4 V and a capacity of 90 mAh g^−1^ [58]. This same reaction is observed for the similar materials with different metals or with different anion units [35,56,59,60].

Overall, polyanion materials are similar to the metal oxides, with reasonable capacities and high voltages. The ability to change the tetrahedron anion units gives them synthetic flexibility. However, the current materials show poor cycling stabilities, making them less viable for rechargeable batteries. Sulfates and silicate are less explored [41]. These materials may lead to better candidates.

### 2.3. Organic Compounds as Cathodes in Na-Ion Batteries

**Figure 4:**
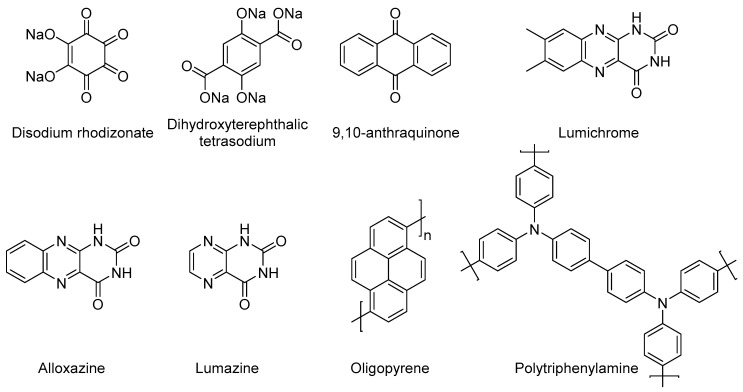
Eight molecules used as organic cathodes in Na-ion batteries. (Adapted from reference [69] with permission).

There has been an increase of research into organic cathode materials, which are cheaper and more eco-friendly [41,61,62,63,64,65,66,67]. These organic materials operate via a reversible Na^+^ ion insertion/extraction mechanism that is accompanied by electrochemically induced transformation of aromatic functional groups. Their performance approaches that of pseudocapacitors, but the use of a sodium anode classifies these systems as batteries [68]. Examples of these functional groups include aromatic imides, quinones, conjugated polymers and aromatic carboxylates (Figure 4).

**Figure 5:**
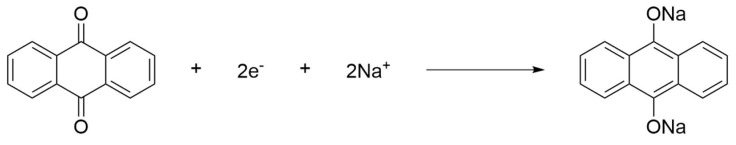
Reduction of 9,10-anthraquinone as model system for organic electrode materials.

An important advantage here is the tunability of the functional groups and/or the structure of the organic compounds [70]. This opens up possibilities for changing the materials’ physical properties such as flexibility and electronic conductivity. The reduction of 9,10-anthraquinone is taken as an example to show the redox reactions of these materials (Figure 5). This reaction gives two phenoxides, which bind the Na^+^ ion [71]. The almost pure ionic bond makes these materials similar to pseudocapacitors. Such systems can achieve high capacities up to 255 mAh g^−1^ at a current density of 50 mA g^−1^ for alloxazine [72]. The capacities are higher than those of metal oxides and polyanions, but come at the cost of a lower voltage range (around 2.2 V for most) and lower stability.

### 2.4. Closing Remarks on Sodium-Ion Batteries

The fate of Na-ion batteries is unclear. The inherent lower gravimetric capacity stays a problem, as does the need for different anode materials. However, sodium has two important features. Firstly, the similar voltage of Na-ion batteries compared to Li-ion batteries will reduce the problems when transiting between the battery types. Secondly, the ability to use the cathode materials already developed for Li-ion batteries will reduce the initial investment required for switching.

## 3. Magnesium-Ion Batteries

Magnesium-ion batteries have an edge over lithium-type batteries since metallic magnesium can be used as the anode (2205 mAh g^−1^), as it is much more stable. It is also more abundant and does not suffer from dendritic growth [73]. The redox potential ($E_{{\mathrm{Mg}}^{2+}/\mathrm{Mg}}$ = −2.37 V) theoretically allows relatively high battery voltages. These advantages of Mg have long been recognized, yet research of Mg-ion batteries is still at a very early stage. While metallic magnesium anodes do not pose a safety risk, they do form a passivated layer of insoluble salt by reacting with many of the commonly used solvents and anions. Other problems include slow intercalation kinetics (due to the high charge density of Mg^2+^) and volume expansions plaguing the systems. Readers interested in these battery types are also referred to the review of Kuang et al. [73].

Thus, both anode and cathode materials must be improved for this battery system. The main eleven categories for the cathode materials are metal selenides [24,74], metal oxides [75,76,77,78,79,80], carbon [81,82,83,84], metal sulfides [85,86,87], Prussian blue [88], Mg-OMS-1 [89], polyoxometalate-(poly)pyrrole [90], MXene [91], metal phosphates [92,93] and magnesium octahedral molecular sieves [94]. Research in anode material is mainly focused on alloys [95,96,97,98]. We will use a selection of these material types to explain magnesium storage mechanisms.

### 3.1. Metal-Oxides as Cathodes in Mg-Ion Batteries

Metal oxides can show two different mechanisms for Mg^2+^ ion storage. The most common mechanism for these materials is the intercalation of Mg^2+^ ions accompanied by a change in the valence state of the metal in the electrode during cycling. This is highly similar to the mechanism for sodium and lithium, but now needs to accommodate the bivalent Mg^2+^ ion. As with Na-ion batteries, α-MnO_2_ has shown remarkable capabilities of Mg^2+^ ion diffusion. However, this material behaves differently with aqueous and non-aqueous electrolytes. For example, the α-MnO_2_/carbon nanotube composite cathode in aqueous electrolyte shows the intercalation mechanism, shown in Equation (6), for Mg^2+^ ion storage [80]. In this mechanism, the Mg^2+^ ion intercalation is accompanied by a change in Mn valence state (Mn^4+^ → Mn^3+^ and Mn^4+^ → Mn^2+^). However, the use of carbon creates the conditions for an additional storage mechanism. The physical adsorption of Mg^2+^ ions forms an electric double layer, where the charge is compensated by the negative ions in the electrolyte solution. In non-aqueous electrolyte, a conversion mechanism is observed. This mechanism type is characterized by the formation of separate phases as seen in Equation (7), where MnO, MnOOH and Mg(OH)_2_ are formed [99]. Mg(OH)_2_, however, is a poor conductor and induces rapid capacity fading during cycling.

(6) $${\mathrm{MnO}}_{2}+{\mathrm{Mg}}^{2+}+2e^{-}\to \mathrm{MnO}+\mathrm{MgO}$$

(7) $$\mathrm{MgMn}O_{2}+x{\mathrm{Mg}}^{2+}+x2e^{-}+H_{2}O\to \left(2x-0.7\right)\mathrm{MnO}+\left(1.7-2x\right)\mathrm{MnOOH}+\left(x+0.15\right)\mathrm{Mg}{\left(\mathrm{OH}\right)}_{2}$$

The intercalation of Mg^2+^ ions has also been observed for various vanadium oxides, such as NaV_3_O_8_ [75,79], NH_4_V_4_O_10_ [76] and H_2_V_3_O_8_ [77]. X-ray photoelectron spectroscopy showed a decrease of V^5+^ during discharge, while the amount of V^4+^ increased. These changes, unlike for manganese, are still observed in non-aqueous electrolyte, indicating that this pathway is more favorable for vanadium oxide cathodes.

The materials generally reach reasonable capacities. For example, using δ-MnO_2_ cathodes yields an initial capacity of 250 mAh g^−1^ and a voltage of 1.0 V at a current density of 100 mA g^−1^ in aqueous electrolyte [78]. Furthermore, this material shows good rate capabilities, as the capacity is retained by 84%, 60%, 54% and 36% upon increase of the current density by 2, 5, 10 and 20 times, respectively. Finally, the material shows excellent cycling stability retaining 84% of the initial capacity (75 mAh g^−1^) after 1500 charge/discharge cycles at a current density of 3 A g^−1^.

### 3.2. Metal Selenides as Cathodes in Mg-Ion Batteries

There are two recent publications utilizing metal selenides as cathodes for magnesium-ion batteries: one using a Mg^2+^/Li^+^ hybrid battery [24] and one testing the material in both Li-ion and Mg-ion batteries [74]. The Mg^2+^/Li hybrid battery uses a magnesium anode and adds 0.5 M LiCl to the electrolyte. The electrolyte used in the system is an all-phenyl-complex (APC), containing 0.2 M AlCl_3_, 0.4 M PhMgCl and anhydrous tetrahydrofuran. Due to the lithium additive, the mechanism of the hybrid battery is based on reversible intercalation/deintercalation of Li^+^ ions. These types of batteries would provide the advantages of both Li-ion batteries and Mg-ion batteries: fast intercalation kinetics (Li-ion), no dendrite formation (Mg-ion), increased safety (Mg-ion) and low-cost (Mg-ion). Furthermore, since the intercalation mechanism is based on Li-ions, the scope of applicable cathode material increases significantly.

The second paper uses novel hexagonal NbSe_2_ as a cathode material for Mg-ion batteries [74]. Using ex-situ X-ray photoelectron spectroscopy, the authors found that Nb^4+^ is reduced to Nb^3+^ during the discharge process, while the Se^2−^ remains unchanged, see Equation (8). This means that the valence state of the metal in the cathode material changes upon intercalation of the Mg^2+^ ions, which was previously observed for metal oxides as well. Furthermore, a strong peak for Cl is observed in X-ray photoelectron spectroscopy, which is attributed to precipitated MgCl_2_ from the APC electrolyte.

(8) $${\mathrm{Mg}}^{2+}+2e^{-}+2{\mathrm{NbSe}}_{2}\to \mathrm{Mg}{\left({\mathrm{NbSe}}_{2}\right)}_{2}$$

The best results were obtained by the Mg^2+^/Li^+^ hybrid battery, showing an initial capacity of 204 mAh g^−1^ at a current density of 100 mA g^−1^ and a voltage of 1.27 V [24]. However, in the second cycle, the capacity had decreased to 160 mAh g^−1^. The cycling stability of the hybrid batteries was tested for 100 cycles at a current density of 200 mA g^−1^, showing 80% capacity retention (110–89 mAh g^−1^) from the 3rd to 100th cycle. The combination of low capacity (unstable till the 3rd cycle) and the use of expensive lithium, makes it where this material is not a viable option for large grid storage. Metal/lithium hybrid batteries are promising for a transition period. They increase the safety of Li-ion batteries, while reducing the Li used and combine this with the benefits of the metal anode. Yet these systems are no long-term solution.

### 3.3. Metal Sulfides as Cathodes in Mg-Ion Batteries

The mechanism for metal sulfide cathodes is similar to that of metal oxides [100]. However, the co-intercalation mechanism was observed for MoS_2_ as cathode material [86]. Here the Mg^2+^ ions complexes with dimethyl ether (DME), forming ([Mg(DME)_3_]^2+^ and results in fast intercalation kinetics. In the solvated ions ([Mg(DME)_3_]^2+^) co-intercalation, the DME molecules form a complex with Mg^2+^ ions. These complexes weaken the interaction between Mg^2+^ ions and the 2D host (MoS_2_) layers. This increases the intercalation kinetics of Mg^2+^ ions. Once inside the cathode lattice, the Mg^2+^ ion reacts with the MoS_2_. This reaction is the same as observed in metal oxides and metal selenides [74,80]. In this mechanism, the Mo^4+^ in the cathode is reduced by the electrons and not the Mg^2+^ ions, see Equation (9). In this reaction the Mo^4+^ is reduced to Mo^2+^, while the S^2−^ remains unchanged. Co-intercalation between ether-solvents and Mg^2+^ ions may be a suitable route to overcome the sluggish intercalation kinetics in multivalent ion-based batteries. This co-intercalation was already shown to improve the kinetics for carbon-based cathodes as well [81].

(9) $${\mathrm{Mg}}^{2+}+2e^{-}+{\mathrm{MoS}}_{2}\to {\mathrm{MgMoS}}_{2}$$

The best results for metal-sulfide based cathodes were obtained with a graphene wrapped VS_2_ cathode, magnesium anode and all-phenyl-complex electrolyte with Li as additive [87]. This system circumvents the sluggish kinetics of Mg^2+^ ion insertion by using a Mg/Li hybrid battery. The battery has a capacity of 146 mAh g^−1^ and a voltage of 2 V at a current density of 900 mA g^−1^. Furthermore, it shows high stability for 10,000 cycles.

### 3.4. Carbon-Based Materials as both Anodes and Cathodes in Mg-Ion Batteries

In general, the intercalation of Mg^2+^ ions into graphite layers is energetically unfavorable. However, when this problem is overcome, carbon-based anodes and cathodes can be used for Mg-ion batteries. The anode will function similar to that in sodium and lithium. The carbon cathode will use the possibility of the metallic magnesium anode. After the oxidation of the magnesium at the anode, Mg^2+^ ions (0.72 Å) insert between the cathode carbon layers and are reduced [82,84]. The stabilization of the metallic magnesium by the carbon layers reduces the energy state, resulting in the overall energy gain of the system.

**Figure 6:**
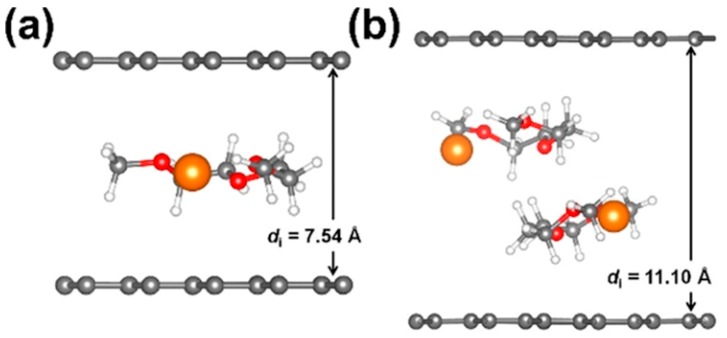
Model of a Mg^2+^–DEGDME cointercalated graphite: (**a**) single- and (**b**) double-layer structures. Orange, white, gray, and red spheres represent Mg, H, C, and O atoms, respectively. d_i_ represents the intercalant gallery height (figure reproduced from reference [81] with permission.).

Recently, Mg^2+^ complexes were shown to co-intercalate into graphite [81]. In the cathode, the Mg^2+^ ion is reduced and will form some metallic magnesium in the cathode material. To clarify the co-intercalation mechanism of graphite with Mg^2+^ ions, structural changes in the natural graphite were investigated through ex situ X-ray diffraction analysis at various states during charge and discharge. Comparing the experimental results with density functional theory (DFT) calculations, shows that Mg^2+^-diethylene glycol dimethyl ether (DEGDME) complexes are double stacked (Figure 6) in the fully discharged graphite. The diffusion of the Mg-ions is increased by complexation with DEGDME. In the diffusion process, a molecule of DEGDME surrounds a Mg^2+^ ion and moves parallel to the graphite surface. This lowers the diffusion barrier of Mg^2+^ ions and the lowered diffusion barrier (1.5 × 10^−8^ cm^2^ s^−1^) is comparable to that of Li^+^ ions in graphite (1.8 × 10^−9^ cm^2^ s^−1^).

**Figure 7:**
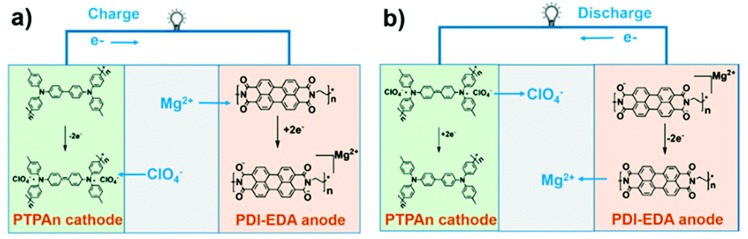
Illustration of the working mechanism of a Mg-ion battery based on perylene diimideethylene diamine (PDI-EDA) and polytriphenylamine (PTPAn) showing the charging process (**a**) and the discharging process (**b**). (From reference [83] with permission.).

Another mechanism that was proposed for carbon-based materials is the dual-ion energy storage mechanism [83]. This pertains to systems using carbon-based material as both anode and cathode. Perylene diimideethylene diamine (PDI-EDA) and polytriphenylamine (PTPAn) were used as anode and cathode, respectively and an anhydrous acetonitrile solution of magnesium perchlorate was used as electrolyte. During the charging process, Mg^2+^ ions associate with the reduced carbonyl groups of PDI-EDA, while the ClO_4_^−^ anions can be bounded with the radical cations of the PTPAn cathode (Figure 7). In the discharging step, Mg^2+^ and ClO_4_^−^ ions are released from PDI-EDA and PTPAn, respectively, to fulfill the cycling processes of the magnesium-ion based organic secondary battery. This system functions as a capacitor, rather than a battery. It does not have a metallic magnesium anode and rather than the magnesium reacting with the cathode material, it is electrostatic interaction that hold it in the material.

The best results were obtained using poly(hexyl viologen dichloride) as cathode, magnesium as anode and an all-phenyl-complex electrolyte, containing 1 M AlCl_3_, 2 M PhMgCl and anhydrous THF solvent [84]. This system delivered a capacity of 171 mAh g^−1^ at a current density of 17 mA g^−1^ and shows good cycling stability after initial stabilization. The capacity decreases from 192 mAh g^−1^ to 136 mAh g^−1^ during second discharge process but then increases to 171 mAh g^−1^. The system delivers a voltage of 1.33 V.

### 3.5. Metal Alloys as Anodes in Mg-Ion Batteries

As previously mentioned, the cathode is not the only thing that has to be improved to make magnesium-ion based batteries viable. Metallic magnesium can react with all known electrolytes based on oxidatively stable solvents and salts, such as Mg(ClO_4_)_2_ and Mg bis(trifluoromethanesulfonimide), Mg(TFSI)_2_) [85]. This reaction forms a passivating layer at its surface and preventing the reversible plating and stripping of Mg. One can avoid this layer formation by using magnesium-metal alloys. The materials explored for this application are bismuth nanocrystals, a magnesium/tin alloy [96,98], bismuth/tin alloy [95] and a biphase bismuth-tin film [97]. Out of these materials, only the bismuth nanocrystals have been tested as anode in a full battery cell. The other materials were tested for their magnesium storage capacities by using metallic magnesium as counter electrode.

The system that tested the bismuth nanocrystals as anode uses Mo_6_S_8_ as cathode material. They propose their colloidal bismuth nanocrystals could serve as a new model anode and studied the Mg^2+^ ion intercalation mechanism of this material [85]. Specifically, the intercalation of Mg^2+^ ions within Bi nanocrystals takes place through an alloying mechanism, leading to the simultaneous formation of α-Mg_3_Bi_2_ and β-Mg_3_Bi_2_, as in Equation (10). The reaction at the cathode is the same as for metal-sulfide cathodes, see Equation (11).

(10) $${\mathrm{Mg}}_{3}{\mathrm{Bi}}_{2}\to 3{\mathrm{Mg}}^{2+}+\mathrm{Bi}+6e^{-}$$

(11) $${\mathrm{Mo}}_{6}S_{8}+3{\mathrm{Mg}}^{2+}+6e^{-}\to {\mathrm{Mg}}_{3}{\mathrm{Mo}}_{6}S_{8}$$

The other alloy materials were tested for their magnesium storage/release capabilities [95,96,97,98]. The materials are tested with magnesium as a counter/reference electrode, effectively making them the cathode in these systems. All these materials follow an Mg alloying/dealloying mechanism upon discharge/charge, respectively. The alloys that were observed in the material are Mg_2_Sn and Mg_3_Bi_2_. Since the alloying is reversible the Mg alloys can be used as an alternative for pure magnesium as anodes in Mg-ion batteries. This would allow for a wider range of usable electrolytes since the alloys do not appear to form a passivation layer.

The best results were obtained using Bi nanocrystals as anode, Mo_6_S_8_ as cathode and 1 M solution of Mg(TFSI)_2_ in diglyme as electrolyte. However, these materials are incomparable as the bismuth nanocrystals are the only material tested as anode in a full cell set up. The other materials are only tested on their reversible magnetization/demagnetization mechanism. The system shows a stable capacity of 325 mAh g^−1^ over at least 150 cycles at a current density of 770 mA g^−1^ and delivers a voltage of ~1.2 V. This paper is also an example as it uses a benchmark cathode (Chevrel-phase Mo_6_S_8_) and a common electrolyte (Mg(TFSI)_2_ in diglyme) to test the electrochemical properties of their new anode material. If everyone in the field of battery chemistry was to use model systems while testing their material, understanding the added value of the new material would be significantly easier. Using magnesium metal alloys as anode material would be an interesting route to explore, since it negates the formation of a passivation layer on the anode, which is one of the major issues for magnesium anodes.

### 3.6. Closing Remarks on Magnesium-Ion Batteries

As a whole, Mg-ion batteries are a promising alternative for Li-ion ones. Both cathodes and anodes have been researched extensively, leading to the discovery of magnesium-metal alloys as potential anodes. Using alloys would make Mg-ion batteries compatible with a variety of electrolytes that are unusable when using metallic magnesium. Both anode and cathode show high cycling stability and most materials show good rate capabilities. The biggest edge that lithium batteries have over Mg-ion batteries is the high voltage they deliver. The voltage of an average Mg-ion battery lies between 1–2 V, which is 2–3 times lower than that of a Li-ion one.

## 4. Zinc-Ion Batteries

Rechargeable zinc-ion batteries are one of the most promising alternatives for grid energy storage. They are safer than lead-acid batteries [101]. They have many advantages, though both the theoretical gravimetric of metallic zinc (820 mAh g^−1^) and its redox potential ($E_{{\mathrm{Zn}}^{2+}/\mathrm{Zn}}$ = −0.76 V) are low [14]. One of these is the relatively easy dissolution and deposition compared to, for example, magnesium. Metallic Zn anodes are more stable than Mg ones, but they do suffer from dendrite formation [102]. The relative anode stability results in most of the research focusing on the cathode and electrolyte. The cathode materials can be divided into different categories; metal oxides [103,104,105,106,107,108,109], metal sulfides [110,111,112], vanadium phosphates [113,114,115], carbon [116,117,118,119,120], MoO_2_/Mo_2_N heterostructure nanobelts [121] and potassium copper hexacyanoferrate [122]. Here we focus on those papers that explain the mechanism for each material type. Using these systems we will try to explain the workings of a batteries wherein zinc is the energy carrier. For more information on these battery types see the review by Fang et al. [123].

### 4.1. Metal Oxides as Cathodes in Zn-Ion Batteries

As with the sodium and magnesium, the two main metal oxides cathodes for Zn-ion batteries are manganese and vanadium oxides. Yet their storage mechanisms differ: Vanadium oxide intercalates the Zn^2+^ ion into its lattice and the reduction of vanadium accommodates for the Zn^2+^ ions binding to the oxygen in the cathode material [106,108]. Clear evidence of this mechanism was observed via X-ray diffraction with a (NH_4_)_2_V_10_O_25_**⋅**8H_2_O cathode. Here, the X-ray diffraction peaks showed the formation of Zn_3_(OH)_2_V_2_O_7_**⋅**8H_2_O during discharge. The intensity of these peaks increased until the system was completely discharged and diminished during charging. Further evidence was found with X-ray photoelectron spectroscopy, which showed an increase in V^4+^ during discharge at the cost of V^5+^. This was reversed during charging and shows the highly reversible transformations between V^5+^ and V^4+^ during the cycling.

**Figure 8:**
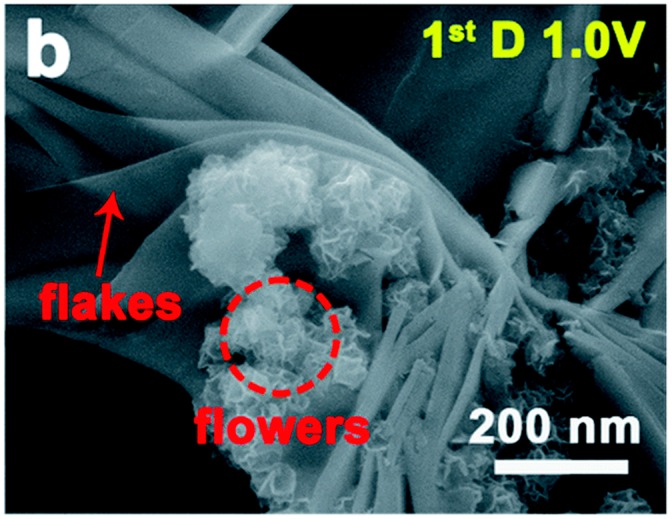
SEM image of Mn_3_O_4_ nanoflowers after discharge, showing the large flakes of ZnSO_4_**⋅**3Zn(OH)_2_**⋅**5H_2_O. (From reference [103] with permission).

The manganese oxides also store the electrons by reducing the manganese in the crystal lattice. However, it does not follow an intercalation mechanism, but rather a chemical conversion mechanism. This involves the formation of zinc sulfate hydroxide hydrate (ZnSO_4_**⋅**3Zn(OH)_2_**⋅**5H_2_O), which forms large flakes on the manganese oxide [103,109]. The existence of the flakes is confirmed by ex situ X-ray diffraction, X-ray photoelectron spectroscopy and SEM at fully charged and discharged Mn_3_O_4_ nanoflowers. Both a MnO and ZnSO_4_⋅3Zn(OH)_2_**⋅**5H_2_O phase were observed after full discharge. Electrons from the anode are stored during the discharge via the reduction of Mn^3+^ (Mn_3_O_4_) to Mn^2+^ (MnO), see Equation (12). The surplus oxygen from the transition of Mn_3_O_4_ to MnO reacts with the ZnSO_4_ and H_2_O to form the ZnSO_4_⋅3Zn(OH)_2_**⋅**5H_2_O, see Equation (13). The resulting flakes are large enough to be observed with SEM (Figure 8). During charging, both phases disappear and the Mn_3_O_4_ phase reemerges, suggesting good reversibility of the cathode.

(12) $${\mathrm{Mn}}_{3}O_{4}+2e^{-}+H_{2}O\to 3\mathrm{MnO}+2{\mathrm{OH}}^{-}$$

(13) $$4{\mathrm{ZnSO}}_{4}+5H_{2}O+6{\mathrm{OH}}^{-}\to {\mathrm{ZnSO}}_{4}\cdot 3\mathrm{Zn}{\left(\mathrm{OH}\right)}_{2}\cdot 5H_{2}O$$

The possibilities of these materials are showcased with the Zn//Zn(CF_3_SO_3_)_2_//V_6_O_13_·*n*H_2_O battery [104]. This system delivers an initial discharge capacity of 300 mAh g^−1^, at a current density of 5 A g^−1^. Furthermore, after 1000 cycles a capacity of 262 mAh g^−1^ is maintained. Overall, metal oxides are promising cathode material for Zn-ion batteries, owing to their high cycle stability (4000 cycles) and high rate-capabilities (20 A g^−1^) [106]. They also have capacities ranging from 57 to 396 mAh g^−1^. Furthermore, there is also a good understanding of the mechanism, making the rational design of cathode material significantly easier. If the capacity of these systems could be increased, they would become a competitive alternative to lithium-based batteries, especially for large grid energy storage.

### 4.2. Metal Sulfides as Cathodes in Zn-Ion Batteries

Metal sulfide cathodes for Zn-ion batteries can undergo multiple different charging mechanisms. Some follow the simple intercalation mechanism, with the reduction of the metal in the cathode [111,112]. Defects and sulfur vacancies in the metal sulfide enhances this intercalation. However, other mechanisms have also been observed, for example when using VS_4_ as cathode [110]. With this material, a combination of two reactions can be observed. The first is the intercalation reaction of Zn^2+^ ions with the VS_4_, as in Equation (14). However, recharging the material results in the partial conversion of VS_4_ to Zn_3_(OH)_2_V_2_O_7_**⋅**2H_2_O (mind that vanadium is now V^5+^) and orthorhombic sulfur, see Equation (15). The authors note that more complicated conversions are likely to take place during discharge and do not yet fully elucidate the discharge mechanisms. One possibility is the formation of a sulfur cathode type battery. Although the mechanism is uncertain, good cycling stability for 170 cycles are observed. Furthermore, the Zn//Zn(CF_3_SO_3_)_2_//VS_4_ battery delivers a lower capacity (180 mAh g^−1^) than the metal oxide batteries, but has a high capacity retention.

(14) $${\mathrm{Zn}}^{2+}+2e^{-}+{\mathrm{VS}}_{4}\to {\mathrm{ZnVS}}_{4}$$

(15) $$2{\mathrm{VS}}_{4}+11H_{2}O+3{\mathrm{Zn}}^{2+}\to {\mathrm{Zn}}_{3}{\left(\mathrm{OH}\right)}_{2}V_{2}O_{7}+8S+16H^{+}+10e^{-}$$

Overall, the metal sulfides give reasonable capacities and high cycle stability and rate capacity. While unusual storage mechanisms are not observed for all materials, these can be a potential disadvantage (formation of unexpected phases) or advantage (high capacities) for these materials.

### 4.3. Polyanion Compounds as Cathodes in Zn-Ion Batteries

Currently, polyanion Zn-ion batteries only use vanadium phosphates type materials as the cathodes. These follow a similar intercalation mechanism as the Na-ion polyanion batteries, even as far as having Na^+^ ions incorporated in the material [113,114]. Again, the V^3+^/V^4^+ redox pair is essential for the battery, as demonstrated by the Na_3_V_2_(PO_4_)_2_F_3_ cathode. The Na^+^ ions are extracted during charging and some of the V^3+^ is oxidized to V^4+^, according to the X-ray photoelectron spectroscopy study, as shown in Equation (16). During discharge, instead of the Na-ions intercalating back into the material, Zn^2+^ ion inserts and forms Zn_0.5_Na_2_V_2_(PO_4_)_2_F_3_, see Equation (17). Alternatively, both Na^+^ and Zn^2+^ ions co-intercalate into Na_3_V_2_(PO_4_)_3_, behaving more like a Zn/Na hybrid battery. The hybrid system also results in a good initial discharge capacity of 101 mAh g^−1^ at a current density of 500 mA g^−1^ and delivers a voltage of 1.23 V at 50 mA g^−1^. It does lose a significant amount of this capacity (76 mAh g^−1^) after 200 cycles.

(16) $${\mathrm{Na}}_{3}V_{2}{\left({\mathrm{PO}}_{4}\right)}_{2}F_{3}\to {\mathrm{Na}}_{2}V_{2}{\left({\mathrm{PO}}_{4}\right)}_{2}F_{3}+{\mathrm{Na}}^{+}+e^{-}$$

(17) $$2{\mathrm{Na}}_{2}V_{2}{\left({\mathrm{PO}}_{4}\right)}_{2}F_{3}+{\mathrm{Zn}}^{2+}+2e^{-}\to \mathrm{Zn}{\left({\mathrm{Na}}_{2}V_{2}{\left({\mathrm{PO}}_{4}\right)}_{2}F_{3}\right)}_{2}$$

These material are currently lacking compared to the metal-oxides and metal sulfide cathodes. They underperform in capacity, cycle stability and high rate-capabilities, while using similar elements. Maybe with enough time, these materials can perform better, but a serious breakthrough is necessary to overtake the metal-oxides and metal-sulfides.

### 4.4. Carbon-Based Materials as Cathodes in Zn-Ion Batteries

The charge/discharge mechanism for carbon materials for Zn^2+^ ion storage mechanisms follows the rocking chair mechanism also observed in Li-ion batteries [116]. However, the capacity can be enhanced by functionalizing the carbon, forming a pseudocapacitor/battery hybrid. Herein, the functional groups are reduced/oxidized during charge/discharge, storing additional charge [117,120]. For instance, using a polydopamine cathode, the reaction mechanism involves the redox reaction between catechol and ortho-quinone accompanied by Zn^2+^ ion adsorption and desorption [120]. Furthermore, the mechanism for polyaniline as cathode material has also been described [117]. During discharge, =NH^+^– groups are reduced to –NH–. At the same time, the =N– in polyaniline is reduced to –N^−^–, which can bind the Zn^2+^ ions via electrostatic interactions [117]. After completion of the charge process, the reactions are reversed and the material is returned to its original state. This leads to the Zn^2+^ ions that interacted with the negatively charged groups on the polyaniline being removed from the cathode material.

The best results were obtained using a polyaniline cathode, zinc anode and 2 M ZnCl_2_ and 3 M NH_4_Cl aqueous electrolyte [118]. The system delivers an initial capacity of 122 mAh g^−1^ at a current density of 8 A g^−1^, after 1000 cycles at this current density ~100% of the capacity is retained. Furthermore, the aqueous Zn-ion battery works well under bending, folding and twisting, making it an interesting material for flexible electronics. Carbon materials show excellent cycle stabilities as well as good rate capabilities. However, carbon suffers from low theoretical capacity, making the material not very appealing for small electronic devices. Carbon cathodes could be used for grid storage applications, where capacity is less crucial.

### 4.5. Closing Remarks on Zinc-Ion Batteries

As a whole, Zn-ion batteries are unlikely to replace Li-ion ones. They deliver a low voltage in the range of 1–2 V and have the lowest theoretical gravimetric capacity of all the described materials. However, aqueous Zn-ion batteries are among the safest battery systems and with the emerging flexible electronics, the need for flexible energy storage devices increases. For this application, Zn-ion batteries are a fitting choice as a lot of research has already been performed on flexible batteries [118,119,120,124,125,126].

## 5. Aluminum-Ion Batteries

Aluminum battery systems are a promising alternative for Li-ion batteries due to their low cost and high abundance [14]. Furthermore, aluminum is the third richest element in the earth’s crust (7.45%) and its redox reaction ($E_{{\mathrm{Al}}^{3+}/\mathrm{Al}}$ = −1.66 V) involves three electrons, which means a potential high charge-storage capacity. The theoretical volumetric capacity of aluminum (8056 mAh cm^−3^) is about four times higher than lithium (2042 mAh cm^−3^) and its gravimetric capacity (2981 mAh g^−1^) is comparable to lithium (3861 mAh g^−1^) [14]. The most researched component for aluminum-ion batteries is the cathode. The research can be divided into six groups based on the type of material used for the cathode: metal oxides [127,128,129,130,131,132], metal sulfides [133,134,135,136,137], carbon [138,139], metal selenides [14,140] metal phosphides [141,142] and metal phosphite [143]. Here we highlight those works that focus on the function of the material. Based on these, we explain the workings of a batteries using aluminum as the energy carrier.

### 5.1. Metal Alloys as Cathodes in Al-Ion Batteries

The mechanism of the Al-ion storage mechanism depends on the cathode material. For metal oxides, the trivalent aluminum ions react with the metal oxide, forming Al_x_M_y_O_z_ or even Al_2_O_3_ [127,129,130]. In these systems, the aluminum metal at the anode side releases three electrons and the resulting Al^3+^ ion combines with AlCl_4_^−^ ions to form Al_2_Cl_7_^−^ ions, see Equation (18). Meanwhile, on the cathode side, a dissociation reaction of Al_2_Cl_7_^−^ ions generating Al^3+^ and AlCl_4_^−^ ions takes place. The released Al^3+^ ion simultaneously participates in the intercalation process in the cathode, resulting in Al_x_M_y_O_z_, as shown in Equation (19).

(18) $$\mathrm{Al}+7{\mathrm{AlCl}}_{4}^{-}\to 4{\mathrm{Al}}_{2}{\mathrm{Cl}}_{7}^{-}+3e^{-}$$

(19) $${\mathrm{AlV}}_{3}O_{9}+x{\mathrm{Al}}^{3+}+3e^{-}\to {\mathrm{Al}}_{3+x-y}V_{3}O_{9-\frac{3}{2}y}+\frac{y}{2}{\mathrm{Al}}_{2}O_{3}$$

Thus effectively, the Al^3+^ ions released by the anode travel to the cathode material where they bind to the oxygen [127]. This leads to the V^5+^ in the cathode framework being reduced to V^4+^ and V^3+^ by the electrons. However, this reaction has shown to be not completely reversible, leading to a loss in capacity due to accumulation of aluminum in the cathode material. ZnO was also tested as cathode in Al-ion batteries [129]. Upon repeated intercalation/deintercalation of Al^3+^ ions, a decrease in capacity was observed. This capacity fading was correlated to a decrease in crystallinity of their cathode material.

The best results were obtained using SnO_2_/C nanocomposite as cathode, aluminum as anode and AlCl_3_ in 1-ethyl-3-methylimidazolium chloride (1.3:1 mol) ionic liquid electrolyte [128]. The system has a capacity of 370 mAh g^−1^ at a current density of 50 mA g^−1^ and delivers a voltage of 1.95 V. Furthermore, the material shows no capacity fading after 20,000 cycles at a current density of 2 A g^−1^.

Overall, metal oxide cathodes are promising material for Al-ion batteries. However, many materials are unstable or undergo irreversible reactions with Al^3+^ ions. In order to improve metal oxides as cathode materials for Al-ion batteries, the material must be stable upon repeated Al^3+^ ion intercalation/deintercalation. The material also needs to bind aluminum in a way where the reaction is reversible to improve the cycle stability and negate the accumulation of aluminum in the cathode material.

### 5.2. Carbon-Based Materials as Cathodes in Al-Ion Batteries

**Figure 9:**
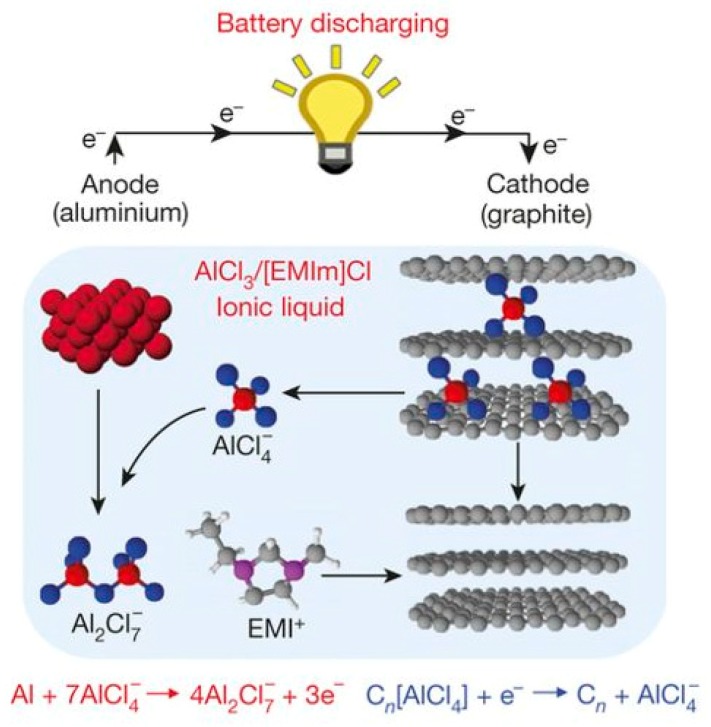
Illustration of the processes occurring in an Al/graphite cell during discharge. (From reference [144] with permission).

The mechanism of carbon-based cathodes differs from that of metal oxides because the carbon materials undergo intercalation of AlCl_4_^−^, rather than the Al^3+^ ions. During the charge of the battery, Al is deposited accompanied by the release of AlCl_4_^−^ species on the metal anode and the intercalation of AlCl_4_^−^ species on the graphite cathode; during the discharge of the battery, Al is dissolved with the formation of Al_2_Cl_7_^−^ species in the anode and the AlCl_4_^−^ ion is released at the cathode (Figure 9) [144]. However, since for aluminum an anion is intercalated rather than the metal cation, the mechanism is inverse. Previously the ions would intercalate in the carbon during discharge. For Al-ion batteries this takes place during discharge, since the AlCl_4_^−^ ions are intercalating and reduction of the carbon leads to electrostatic repulsion.

The overall electrochemical process involves the reversible deposition/dissolution of Al^3+^ ions from the metal anode, and the intercalation/de-intercalation of AlCl_4_^−^ ions in the graphite cathode. Zhang et al. give a detailed overview of different carbon materials and the correlated intercalation mechanism [145]. It is generally believed that the structural quality of graphitic carbons assists in improving the performance of Al-ion batteries [146,147,148]. However, a different approach was also reported, where mesoporous reduced graphene oxide was designed and used as cathode to improve the performance of Al-ion batteries [139]. The material shows electrochemical performance comparable to that of material with much larger surface areas.

The best results were obtained using nanosheet-bricked porous graphite as cathode, aluminum as anode and AlCl_3_ in 1-ethyl-3-methylimidazolium chloride (1:1.3 mol) as ionic liquid electrolyte [138]. The system has a capacity of 104 mAh g^−1^ at a current density of 10 A g^−1^. Furthermore, the systems retains nearly 100% capacity over 3000 cycles even at this high current density.

Overall, carbon materials are promising as cathodes in Al-ion batteries due to their high cycling stability and stability over a big range of current densities. However, the gravimetric capacity of carbon is significantly lower than that of other materials, so using carbon as cathode material makes it nigh impossible to obtain batteries with a high capacity.

### 5.3. Metal Sulfides as Cathodes in Al-Ion Batteries

Most metal sulfide-based cathodes (e.g., Co_3_S_4_ [133], VS_2_ [134], MoS_2_ [135], NiCo_2_S_4_ [136]) follow the same intercalation mechanism as metal oxides. However, using tin sulfide cathodes enables the intercalation mechanism of chloroaluminate anions rather than the Al^3+^ ions, see Equation (18) [137]. In the electrochemical process during charge/discharge, Sn^2+^ is reduced to metallic tin after discharge, whereas the S^2−^ was oxidized to S^6+^ during charge, as in Equation (20).

(20) $$\mathrm{SnS}{\left[{\mathrm{AlCl}}_{4}\right]}_{n}+ne^{-}\to \mathrm{SnS}+n{\mathrm{AlCl}}_{4}^{-}$$

The reaction shown in Equation (22) takes place during discharge. This is why the oxidation state of sulfur changes from 6+ to 2−. The paper does not go into detail as to why their material shows chloroaluminate anion intercalation rather than the Al^3+^ intercalation commonly observed for metal sulfides [133]. It could be because the electronic structure of the tin is similar to that of carbon, which shows the chloroaluminate anion intercalation [134,145]. Another possible reason could be the porosity [137]. For carbon, the electrochemical properties are correlated to the surface area [139,146,147,148]. Since this is a porous metal sulfide structure, rather than micro- or nanostructures, it could be that the intercalation of the larger chloroaluminate ions is faster than the dissociation of AlCl_7_^−^ ions into AlCl_4_^−^ and Al^3+^ ions.

The best results were obtained using the SnS cathode, aluminum anode and AlCl_3_ in 1-ethyl-3-methylimidazolium chloride (1.3:1 mol) ionic liquid electrolyte. The system has a capacity of 406 mAh g^−1^ at a current density of 100 mA g^−1^, delivering a voltage of 2.4 V. After 500 cycles, capacity had decreased to 281 mAh g^−1^, which means the capacity decay rate is only 0.02% per cycle.

Overall, metal sulfides are promising materials for Al-ion batteries, delivering high initial discharge capacities and Coulombic efficiencies. However, the metal sulfide cathodes generally suffer from fast capacity decrease. Furthermore, the current densities for charge/discharge are still inferior to those for lithium-ion batteries. The rate performance of Al-ion batteries is influenced by the solid phase diffusion of Al^3+^ in the cathode, which limits any increase in the charge/discharge current density [133]. To make Al-ion batteries a viable alternative for Li-ion batteries, the material needs to have an excellent long-term cycling stability, as well as a better solid phase diffusion of Al^3+^ ions to increase the performance at higher current densities.

### 5.4. Metal Selenides as Cathodes in Al-Ion Batteries

Metal selenide cathode are electrochemically similar to sulfides. This makes sense as selenium is directly below sulfur in the periodic table. There are two articles describing the use of metal selenides as cathodes in Al-ion batteries. The intercalation mechanism is the same as described for the metal sulfide cathodes [14,140]. In one mechanism, the valency of Co^+2^ in the material does not change during charge/discharge, while the Se^2−^ is oxidized to Se^4+^ or Se^6+^ [14]. This means that the mechanism is probably the same as the mechanism of the SnS cathode [137]. The chloroaluminate ions intercalate in the cathode during charging and the Se^2−^ is oxidized. Upon discharge, the reactions can be described with Equations (20) and (21), with SnS replaced by CoSe in this example. In the second report the Co^2+^ is reduced to metallic cobalt, while the change in oxidation state of selenium is not shown. Here the reaction involves incorporation of Al^3+^ ions in the CoSe_2_ lattice, see Equation (21). The Co^2+^ is reduced during discharge while the Al^3+^ ions remains unchanged.

(21) $${\mathrm{CoS}e}_{2}+x{\mathrm{Al}}^{3+}+3xe^{-}\to {\mathrm{Al}}_{x}{\mathrm{Co}}_{y}{\mathrm{Se}}_{2}+\left(1-y\right)\mathrm{Co}$$

The best results were obtained using carbon encapsulated CoSe as cathode, aluminum foil as anode and AlCl_3_ in 1-ethyl-3-methylimidazolium chloride (1.3:1 mol) ionic liquid as electrolyte. The system has a capacity of 427 mAh g^−1^ at a current density of 1 A g^−1^ and delivers a voltage of 2.1 V. However, the material shows fast capacity decay and the charge capacity is significantly higher than the discharge capacity, leading to poor coulombic efficiency.

Overall, metal selenides are an interesting cathode materials for Al-ion batteries. However, current research is lacking and the materials that have been tested show rapid capacity decay. The metal selenides provide a high capacity and excellent rate capability. Metal selenides need to gain increased cycling stability and coulombic efficiency to become a viable option for cathode material. The high toxicity is also a factor.

### 5.5. Metal Phosphides/Phosphites as Cathodes in Al-Ion Batteries

Metal phosphides/phosphites can show both intercalation of Al^3+^ ions as well as AlCl_4_^−^ ions [141,142,143]. The mechanism for the intercalation of Al^3+^ ions is the same as previously described, reduction of metal in the cathode material and binding of the Al^3+^ ions. However, for Cu_3_P as cathode material, a special mechanism is observed. In this mechanism the AlCl_4_^−^ ions intercalate into the cathode material upon charging, oxidizing both copper and phosphor in the cathode material. Upon discharge, Cl^−^ ions deintercalated from the material and partial reduction of P species occurs, while the oxidation states of Cu and Al remain unchanged.

The best results were obtained by using nickel phosphide as cathode. The system shows a stable capacity of 60.9 mAh g^−1^ over at least 3000 cycles at a current density of 200 mA g^−1^. Overall, metal phosphides/phosphites can be a promising material for cathodes in Al-ion batteries owing to their high cycle stability. However, they suffer from rather low capacities. To become a viable option, the capacity must be increased considerably.

### 5.6. Closing Remarks on Aluminum-Ion Batteries

Overall, aluminum is a viable alternative for lithium for energy storage devices. Al-ion batteries show high capacities, high rate capabilities and aluminum is ~10 times cheaper than lithium [14]. However, the cycling stability of Al-ion battery material varies. There is a trade-off between high cycling stability or high capacity. That said, for large scale applications such as grid storage, the capacity is less important than the stability. Here Al-ion batteries are a viable option, since some systems are stable for >3000 cycles, and the material cost can be a determining factor on large scale.

## 6. Metal-Sulfur Batteries

Another alternative to Li-ion batteries that has received a lot of attention are the metal-sulfur (M-S) batteries (lithium-sulfur batteries are also researched, but these fall outside of the scope of this review). These systems promise a high theoretical capacity, thanks to the sulfur cathode (1672 mAh g^−1^; 3459 mAh cm^−3^) [13]. However, the sulfur cathode also brings new challenges in the form of rapid capacity fading due to sulfide dissolution and polysulfide shuttling [149]. This is the largest barrier for these types of batteries. While they are reported for different metals (e.g., lithium, sodium, aluminum, magnesium), the mechanism and research directions are highly similar.

The energy storage mechanism for all metal-sulfur batteries follows a conversion type mechanism [13,150,151,152,153,154]. A simple version of this reaction occurs for lithium, sodium and magnesium [151,152,153,154,155,156,157,158,159,160,161,162,163,164,165,166]. In this mechanism the sulfur in the cathode is reduced and forms a metal sulfide complex with the metal ions from the electrolyte solution, as in Equation (22).

(22) $$M^{x+}+xe^{-}+S\to M_{2}S\;\left(x=1\right)\;\mathrm{or}\;\mathrm{MS}\;\left(x=2\right)\;\mathrm{or}\;M_{2}S_{3}\;\left(x=3\right)$$

For aluminum a different reaction occurs as the reported systems use an ionic liquid electrolyte, leading to different redox chemistry, see Equations (18) and (23).

(23) $$8{\mathrm{Al}}_{2}{\mathrm{Cl}}_{4}^{-}+6e^{-}+3S\to 14{\mathrm{AlCl}}_{4}^{-}+{\mathrm{Al}}_{2}S_{3}$$

There has not been much research into sodium-, magnesium-, zinc- and aluminum-sulfur batteries, since lithium-sulfur is already regarded as an alternative for Li-ion batteries. For the alternative metals, most of the research is towards the encapsulation of sulfur into porous cathode materials. For example, sulfur composite were prepared into sulfur loaded on a flexible carbon fiber cloth [167], sulfur containing copolymer on reduced graphene oxide [168], sulfur on nitrogen doped graphene nanosheets [169], and sulfur in highly nitrogen and sulfur doped nanoporous carbon [170]. The encapsulation of sulfur into the pores of a sucrose-derived carbon powder is especially interesting. The material has a stable reversible capacity of 370 mAh g^−1^ at current density of 1675 mA g^−1^ for 1500 cycles in a sodium-sulfur battery [171].

Sulfur batteries suffer from capacity fading due to sulfide dissolution and polysulfide shuttling [149]. However, these problems do not occur in Al-S batteries, since elemental sulfur, intermediate polysulfides (include S_6_^2−^ and S_4_^2−^ species), and the final reduction product (Al_2_S_3_) have poor solubility in ionic liquid electrolytes [13]. This insolubility together with the insulating nature of these sulfides results in a high kinetic barrier during electrochemical cycling of the Al-S cells. To negate the polysulfide shuttling, various components of the cell were studied, such as the separator [152,166], the cathode material [154,159] and the electrolyte [152,158,166].

## 7. PEST Analysis

The previous section focused on the theoretical and experimental capacities of the different non-lithium batteries. However, other parameters such as cost, safety, current supply, and total reserves, are as important for economic viability. Here we discuss these parameters and their consequences for the various battery types, using a point-based system for comparison. The parameters are based on the **PEST** analysis, which takes into account **P**olitical, **E**conomic, **S**ocio-cultural and **T**echnological aspects.

### 7.1. Political: Availability

Availability is a key parameter for the viability of different batteries. Indeed, most of the other parameters, such as cost and political concerns, are influenced by international reserves. The points given in the final analysis are solely based on the world production and reserves. For lithium the reserves are estimated to be 53 million tons worldwide, with new reserves being found at an annual basis [172]. The biggest reserves are found in South America (27.2 million tons), China (7 million tons) and Australia (5 million tons). However, lithium production remains low with a total production of 43,000 tons against a consumption of 41,500 tons. Battery manufacturing contributes for 46% of the consumption. The impact of availability on the price was evident in China, where the main outside supply came from Australia. Tightening of the lithium export by Australia made the tonnage cost of lithium range between $15,000 to $24,000 in 2017. Limited suppliers will also raise political concerns since no country wants to be completely dependent on a single other nation for their raw material supply.

The reserves of the substitute metals are much larger. Zinc has an estimated reserve of about 1.9 billion tons, with a 13.53 million ton production and 13.93 million ton consumption in 2017 [173]. The most production is located in China, Peru, Australia and the US, while many countries have relatively small production. Assuming all the lithium consumption will be transformed to zinc, with an atomic mass ten times higher, will make an estimate of 180,000 tons of zinc for battery consumption. This will increase the zinc consumption by 1.3%, which will increase the short term zinc price. Magnesium has a larger reserve then zinc, of about 12 billion ton of magnesite and practically endless in seawater and natural brines [174]. The world production in 2017 of magnesite was 27 million tons, which is more than enough to ensure the availability for batteries. Furthermore, this ore is mined in every continent, ensuring easy excess to this mineral. Aluminum is produced on a 60 million ton scale and has a reserve of 55–75 billion ton in bauxite alone [175]. The primary producers are China, Russia, Canada and India, but aluminum is also being produced in many other countries. Sodium has the biggest supply, with an endless reserve in our oceans. It is produced on a massive scale, with a production of 280 million tons annually [176]. While the major producers are China, the US and India, every country with excess to seawater is able to produce salt. In sulfur batteries the metal containing counter electrode is replaced by a sulfur containing electrode. Sulfur itself is a byproduct of fossil fuel refinement and is produced on a massive scale [177]. Since the use of fossil fuels will not diminish in the near future, supply of sulfur will not dwindle. This also means that any country able to extract fossil fuels is able to produce sulfur. The use of sulfur cathodes will reduce the need of transition metals in the battery, which are usually less available then sulfur.

### 7.2. Economic: Cost

The cost of a battery comprises raw materials, labor and equipment costs [178]. The raw materials account for ca. 60% of the costs of producing a battery. From this 60%, 10–15% of the cost is attributed to the anode and electrolyte, which contain the lithium. This is what will be affected by the replacement of lithium by an alternative material. Furthermore, the cathode accounts for ca. 25% of the total battery costs, which can decrease by replacing the expensive metal-oxides or composites by cheap sulfur. Table 1 contains the bulk prices of the metals for the different battery types. These prices are based on the pure metals, except for sodium and lithium which are most commonly sold as NaCl and Li_2_CO_3_. As lithium has the highest price, battery costs should reduce by changing to a different metal. The point distribution only takes the prices of the materials into account and does not contain any price variations associated with the modifications (e.g., different cathode material, labor cost, overhead cost, permits) necessary for non-lithium-based batteries.

### 7.3. Socio-Cultural: Safety

Safety concerns govern any malfunction that can damage humans or their surroundings, for example explosions or combustion. However, toxicology effects are not addressed here since these deserve their own segment. Li-ion batteries are relatively safe and will only malfunction at high temperatures or when badly designed. However, lithium sulfur batteries are less safe, since the metallic lithium anode can violently react with both oxygen and water. The same principle holds for the Na-ion and Na-S batteries due to the similar reactivity of sodium and lithium. For aluminum, zinc and magnesium, both the M-ion and M-S battery contain metallic anodes, but these react less severe with oxygen and water.

### 7.4. Socio-Cultural: Weight

Changing lithium for another metal has a dramatic effect of the final weight of the battery. For sake of easy comparison, we assume that every battery will hold the same amount of charge unrelated to the used ions. Therefore, the mass per charge of the ions can be used to compare the different metals. Li^+^ ions have the lowest mass per charge (6.94 gr/mol of charge), closely followed by the trivalent Al^3+^ ions (8.99 gr/mol of charge), divalent Mg^2+^ ions (12.16 gr/mol of charge), monovalent Na^+^ ions (22.99 gr/mol of charge) and the heaviest being divalent Zn^2+^ ions (32.69 gr/mol of charge). Another consideration is the lower battery weight when the generally metal containing cathodes are substituted by sulfur cathodes in M-S batteries. However, mass increase is only a problem for mobile applications. The lower the mass per charge, the higher the points given in this section.

### 7.5. Socio-Cultural: Toxicity

The toxicity of the batteries is quite difficult to define since the way of exposure can vary. Leaking of the battery can result in oral intake of any of the metals while an ignition or explosion of the battery can result in inhalation of metal particles. The danger of inhalation of metal particles do not vary much between the different metal-based batteries, thus we focus on the immediate danger in the oral uptake of the different metals. While Aluminum comprises about 5% of the earth’s crust, it is not essential to humans and most flora and fauna. Therefore, the normal intake, 2–3 mg of aluminum, is low and is directly excreted from the body. Since the human body only has to handle small doses on a daily basis, a sudden high uptake of the metal will not be excreted and will cause toxic effects in our body [179]. From the five metals, it is the most poisonous. Zinc plays a vital role in our body and a daily uptake of 8–11 mg of zinc is required. A too high ingestion of zinc results in copper deficiency and related symptoms, while getting a lethal dose (LD50 of 3g/kg of zinc) is unlikely since a dose of 400 mg already induces vomiting [180]. Magnesium, like zinc, plays a role in our bodily functions but we require much more of it (±350 mg daily). Higher doses are generally removed by the kidneys, but a concentration of 1.74–2.61 mmol/L in blood serum can become lethal. This is relative to a 5 g daily intake of magnesium [181]. Lithium is used to treat bipolar and other mental disorders. Due to its use as a medicine, much of its toxicity is known. A serum level of 1.5 mmol/L is considered toxic, but mostly likely show symptoms when the person is ill. An ingestion of 5 g of lithium chloride can be lethal [182,183]. The recommended ingestion of sodium in humans is about 3.5 g daily, while consumption can be much higher. Sodium is toxic in large amounts. A serum level of 150 mmol/L is dangerous, which corresponds to an acute ingestion of 60 gr of salt [184]. Sulfur does not pose danger to the human body and replacing metal cathodes by sulfur lowers the toxicity of the total battery. However when ignited, the sulfur dioxide vapors are hazardous. The point distribution of this parameter pertains to the apparent toxicity of the metals. The lower the score the relative more toxic the metal is and vice versa.

### 7.6. Technological: Current Performance

The performance as a key factor has already been described in this review. Table 2 summarizes the type of batteries, different cathode and anodes, capacities, voltages and storage mechanisms. The higher the capacity and voltage, the higher the points given for this parameter.

### 7.7. Technological: Theoretical Performance

We already discussed the maximum theoretical capacities of the different metals in this review. The more points in this area, the higher the theoretical capacity of the battery.

### 7.8. Technological: Stability

A conclusion based on empirical data on the stability of the different battery types is difficult, because of the imbalance in research towards the different battery types. Therefore, we base the stability on the adherent problems of dendrite formation and polysulfide shuttling reported for the batteries. While these issues can be diminished by carefully designing anodes, cathodes and membranes, finding these solutions will diminish their short-term viability. All metal anode containing batteries suffer from dendrite formation, except the ones based on magnesium. Furthermore, sulfur batteries will suffer polysulfide shuttling which lowers their stability. Lower scores in this section are given to batteries which are more unstable anodes and cathodes.

### 7.9. Summary

**Figure 10:**
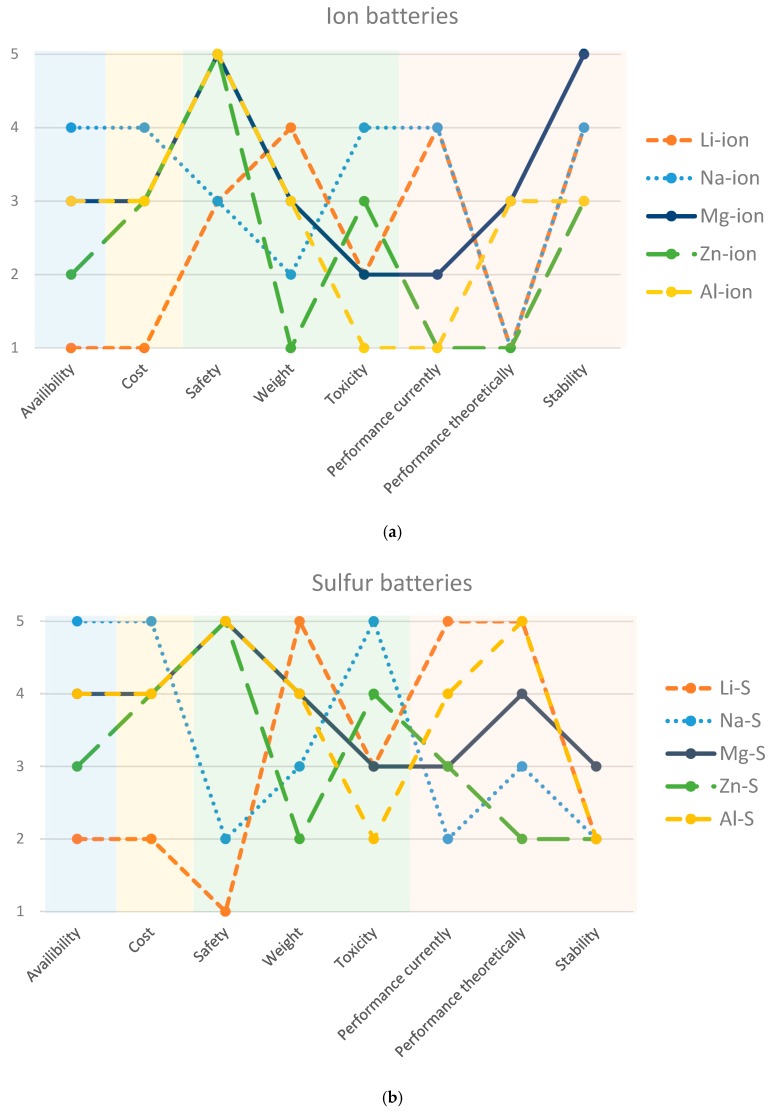
The point distribution outcome for the PEST analysis of the M-ion (**a**) and M-S based batteries (**b**).

Figure 10 summarizes and compares the different parameters for metal-ion batteries (top) and metal-sulfur batteries (bottom). In general, Li batteries score poorly, showing that replacing lithium is not only justified from a technological standpoint, but also socially and politically. Furthermore, every battery has its own pros and cons. The commercialization and success of the batteries depend on which attribute is the most important at a given time. We also see that sulfur batteries score higher than ion batteries, but the technology needed for their implementation is still at an early stage.

## 8. Conclusions and Recommendations

This review highlights the four main alternatives (sodium, magnesium, zinc and aluminum) for lithium in battery applications. The research on these materials is categorized in research towards the cathodes and anodes, and further classified into the different types of materials for these electrodes. Besides the practical aspects, e.g., specific capacity and cycle stability, we also focused on the different storage mechanisms for these materials. In general, metal-based cathodes (e.g., metal oxides, metal sulfides and metal selenides) tend to give high specific capacities in any battery type. Alternatively, carbon cathodes (both as graphite and polymer type) yield lower capacities, although they do sometimes exhibit higher voltages.

Since the chemistry of lithium and sodium is similar, most of the current Li-ion technology can be used for Na-ion batteries. If a sodium metal anode is used, the battery will be less safe, but not using these limits the achievable specific capacity. This is not the only problem of the anodes, with sodium and graphite being incompatible. Currently, Na-ion batteries suffer from a lower capacity and often “poor” cycling stability, especially compared to lithium. This technology also scores low overall in the PEST analysis, with the exception of the cost, availability and current readiness. The biggest future for this type of batteries is in a transition period. The similarities, both in supplied voltage and industrial fabrication, requires a low investment to switch to this battery type. However, the low theoretical capacity will quickly require a different battery type.

Magnesium-ion batteries are a promising alternative for Li-ion batteries. The formation of the passivated layer on the anode seems to be solved with magnesium-metal alloys. Their biggest drawback is their low voltage (2–3 times lower than lithium), requiring a complete overhaul of any battery carrying device. Additionally, the capacity of these batteries is currently low, even further diminished of one includes the supplied voltage. The high stability of these batteries will be a determining factor when the anodes and cathodes are improved for their capacitance.

The low theoretic gravimetric capacity of zinc makes it an unlikely candidates for replacing Li-ion batteries in mobile applications. This is further diminished by their low voltage output and currently average specific capacity. However, aqueous Zn-ion batteries are among the safest battery systems, making them ideal for grid storage. Research should focus on the stability and capacity retention of these systems. Some have reported almost 100% retention, showing the possibilities for these types of batteries.

Theoretically, aluminum is the best option for replacing lithium, with the highest theoretic gravimetric capacity. Furthermore, this battery type has an overall good score in the PEST analysis. The main drawback currently is the low capacity of the reported batteries, with also a visible trade-off between capacity and stability in literature. Current Al-ion batteries can be viable for grid storage, where capacity is less important than stability.

In the more distant future, sulfur based batteries will replace Li-ion batteries. However, the type of metal that will be used is highly dependent on the battery technology at that time. These batteries have by far the highest capacity at high current densities and showcase more futuristic values. The biggest drawback (polysulfide shuttling) of these systems is already identified, improving the aim in the scientific research.

We also identified a different problem within the battery research field. The lack of standardized testing conditions, such as standard cathodes, anodes, electrolyte and current densities, makes direct comparison between different systems difficult. To understand the contribution of new scientific work in this field, such standards are necessary.

## Figures and Tables

**Table 1:** The prices of the bulk elements of the different battery types ^a^.

Element	Cost ($/ton)
Lithium ^b^	17,000
Sodium ^c^	250
Magnesium	2530
Zinc	2740
Aluminum	2300
Sulfur	70

^a^ Average 2018 prices from USGS annual overview. ^b^ As lithium carbonate. ^c^ As soda ash.

**Table 2:** Overview of the electrode types per battery and their corresponding capacities and voltages.

Battery	Component	Material	Capacity (mAh g^−1^)	Voltage (V)	Storage Mechanism	Source
Na-ion	Cathode	Metal oxides	>200	3.5	Intercalation	[53]
		Polyanions	>90	3.4	Intercalation	[58]
		Organic compounds	255	2.2	Pseudo-capacitor	[72]
Mg-ion	Cathode	Metal oxides	250	1.0	Intercalation	[78]
		Metal Selenides	204	1.27	Intercalation	[24]
		Metal sulfides	146	2.0	Intercalation	[87]
	Cathode and anode	Carbon-based	171	1.33	Co-intercalation	[84]
	Anode	Metal alloys	325	1.3	Alloying	[85]
Zn-ion	Cathode	Metal oxides	396	1.4	Intercalation and conversion	[108]
		Metal sulfides	180	1.8	Intercalation and conversion	[110]
		Polyanions	101	1.23	Intercalation	[113]
		Carbon-based	122	1.7	Pseudo-capacitor	[118]
Al-ion	Cathode	Metal alloys	370	1.95	Intercalation	[128]
		Carbon based	104	2.55	Intercalation	[138]
		Metal sulfides	406	2.4	Intercalation	[137]
		Metal selenides	427	2.1	Intercalation	[14]
		Metal phosphides/phosphites	61	2.2	Alloying	[142]
